# Medical Societies

**Published:** 1849-11

**Authors:** 


### Part 4.—Editorial.

### ARTICLE 1.

### MEDICAL SOCIETIES.

"*Central Wabash Medical Society*.---This Society was organized in Williamsport, Indiana, on 11th May last, by adopting a constitution and by-laws, and electing James H. Buell, President; C. V. Jones, Vice-President; Benj. H. Boyd, Secretary; Asa Bigelow, Treasurer; F. Irish, D. K. Hays and S. W. Ritchey, Censors.

"Its members are physicians of Fountain, Warren, and the adjoining counties. Its meetings are held semi-annually on the second Wednesdays of May and November. The object, mutual improvement in the Science of medicine; and its collateral branches. It has been commenced with spirit, and promises success."

The above is furnished for our pages by the intelligent President of the Society, to whom we return our thanks.—From an intimate acquaintance with a number of the gentlemen composing the membership of this association, whom we know to be men of talents, and of the highest respectability in the profession, we shall expect it to be highly interesting and eminently useful.

*Peoria District Medical Society*.---We observe by a notice in the papers that a Semi-Annual Meeting of this spirited Association is to be held in Peoria on the 13th of November inst. From its number of able members, and the zeal with which it has hitherto been supported, we look for a highly beneficial influence to be exerted by each Meeting of this body. Business of the utmost importance to the Profession it is said will be brought before the approaching session.

*Æsculapean Society.*---We a few days ago came in possession of the proceedings of the Annual Meeting of this Society, held in Russellsville, Ills., on the 6th of Nov. 1848, nearly one year ago. We are highly pleased with the minutes, from which we observe our friends read essays, make public addresses, and receive reports from committees. Of the essays our readers will be better able to judge after reading the next few numbers of our Journal. Of the speeches we have no means of giving information. Of the reports of committees, we notice the following:

"The committe 'appointed to take such notice of one ———, M.D., steamer, as in their opinion he deserved,' reported 'said ———, is unworthy of any notice by your committee or society.'"

"Dr. C. M. Hamilton was elected president; Dr. J. R. Wynn 1st Vice-President; Dr. N. Hawley 2d Vice-President; Dr. J. M. Logan Sec'y; Dr. W. B. Norton Treasurer; Drs. S. Thompson, E. C. Banks, and J. R. Craig, Censors."

We hope hereafter to enrich the pages of our Journal with such papers of interest to the profession, as may be read before this Society, and return thanks for the favor already shown us.

We hope all our medical societies will be careful to be represented in the American Medical Association, which is to meet in Cincinnati on the the first Tuesday in May next.—Each organized society is entitled to one delegate for every 10 members, and one for any fractional number over five.

